# Risk of ischemic and hemorrhagic stroke in relation to cold spells in four seasons

**DOI:** 10.1186/s12889-023-15459-4

**Published:** 2023-03-23

**Authors:** Vidmantas Vaičiulis, Jouni J. K. Jaakkola, Ričardas Radišauskas, Abdonas Tamošiūnas, Dalia Lukšienė, Niilo R. I. Ryti

**Affiliations:** 1 Faculty of Public Health, Department of Environmental and Occupational Medicine, Lithuanian University of Health Sciences, Kaunas, Lithuania; 2 Faculty of Public Health, Health Research Institute, Lithuanian University of Health Sciences, Kaunas, Lithuania; 3 Center for Environmental and Respiratory Health Research (CERH), Research Unit of Population Health, Faculty of Medicine, University of Oulu, Oulu, Finland; 4 Biocenter Oulu, University of Oulu, Oulu, Finland; 5 Medical Research Center Oulu, Oulu University Hospital and University of Oulu, Oulu, Finland; 6 Finnish Meteorological Institute, Helsinki, Finland; 7 Laboratory of Population Studies, Institute of Cardiology, Lithuanian University of Health Sciences, Kaunas, Lithuania

**Keywords:** Ischemic stroke, Hemorrhagic stroke, Cold spell, Cold Weather, Seasons

## Abstract

**Background:**

Cold winter weather increases the risk of stroke, but the evidence is scarce on whether the risk increases during season-specific cold weather in the other seasons. The objective of our study was to test the hypothesis of an association between personal cold spells and different types of stroke in the season-specific context, and to formally assess effect modification by age and sex.

**Methods:**

We conducted a case-crossover study of all 5396 confirmed 25–64 years old cases with stroke in the city of Kaunas, Lithuania, 2000–2015. We assigned to each case a one-week hazard period and 15 reference periods of the same calendar days of other study years. A personal cold day was defined for each case with a mean temperature below the fifth percentile of the frequency distribution of daily mean temperatures of the hazard and reference periods. Conditional logistic regression was applied to estimate odds ratios (OR) and 95% confidence intervals (95% CI) representing associations between time- and place-specific cold weather and stroke.

**Results:**

There were positive associations between cold weather and stroke in Kaunas, with each additional cold day during the week before the stroke increases the risk by 3% (OR 1.03; 95% CI 1.00–1.07). The association was present for ischemic stroke (OR 1.05; 95% CI 1.01–1.09) but not hemorrhagic stroke (OR 0.98; 95% CI 0.91–1.06). In the summer, the risk of stroke increased by 8% (OR 1.08; 95% CI 1.00–1.16) per each additional cold day during the hazard period. Age and sex did not modify the effect.

**Conclusions:**

Our findings show that personal cold spells increase the risk of stroke, and this pertains to ischemic stroke specifically. Most importantly, cold weather in the summer season may be a previously unrecognized determinant of stroke.

**Supplementary Information:**

The online version contains supplementary material available at 10.1186/s12889-023-15459-4.

## Introduction

Globally, stroke is the second-leading cause of death and the third-leading cause of death and disability combined [1]. In the European Union (EU), stroke is the leading cause of adult disability [2]. Stroke remains one of the most common causes of death in Lithuania, with mortality rates two times above the EU average [3].

Epidemiological studies in various countries such as the USA, China, Japan, South Korea, Germany and Russia have provided evidence that cold winter weather increases the risk of stroke [4–10]. Emerging evidence shows that not only winter weather [11], but also relatively cold weather defined according to the other seasons, is associated with cardiovascular outcomes like out-of-hospital cardiac arrest [12] and sudden cardiac death [13]. It is unclear whether the same applies to different types of stroke. In addition, the evidence of effect modification by age and sex is conflicting [4, 7, 14–18]. A better understanding of the complex relationships between cold weather and stroke is needed for targeted evidence-based prevention.

The objective of our study was to investigate the associations between personal cold spells and different types of stroke in the season-specific context, and to formally assess effect modification by age and sex. We hypothesized *a priori* that (a) there is an association between cold spells and stroke; (b) the risk of stroke increases when the duration of the cold spell increases; (c) the association is not limited to the winter months; (d) the association is not similar for ischemic and hemorrhagic stroke; (e) age and sex modify the effect of cold spells on stroke.

## Methods

We conducted a case-crossover study of the associations between season-specific cold spells and the risk of stroke in the city of Kaunas, Lithuania, 2000–2015. The study protocol for the WHO MONICA study was approved by the Lithuanian Bioethics Committee (No. 14–27) and the study complies with the Declaration of Helsinki. STROBE guidelines were followed in the reporting.

### Study population

The population-based Kaunas stroke register was used to identify cases of stroke for the study. Patients aged 25–64, who were permanent residents of the city of Kaunas, and who experienced a stroke in Kaunas between 2000 and 2015, were included in this study. Survival or death did not influence eligibility for the study. According to the Lithuanian Statistical Department, the population of the City of Kaunas in the age groups 25–64 was 203,742 in the year 2000, and 198,644 in 2015.

### Definition of stroke and the eligibility criteria for the study

According to the WHO MONICA protocol [19], stroke was defined as “rapidly developed clinical signs of focal (or global) disturbance of cerebral function lasting more than 24 h (unless interrupted by surgery or death) with no apparent cause other than a vascular origin”. Global symptoms apply to patients with coma or subarachnoid hemorrhage without focal neurological signs. Every stroke event had to have its apparent onset within the study period 2000–2015 and had to occur more than 28 days after any previously recorded stroke event in the same subject. Multiple stroke attacks occurring within 28 days from the onset of the symptoms of the first attack were considered as one event.

The Kaunas stroke register was established as part of the WHO MONICA project (Monitoring of Cardiovascular Trends and Determinants) [19]. Medical records including the hospital discharge records, outpatient department care records, autopsy records, medical-legal records, and death certificates were used to identify stroke cases (ICD-9 430–436). The above-mentioned sources were reviewed every three months, except for death certificates which were reviewed monthly.

**Fig. 1:**
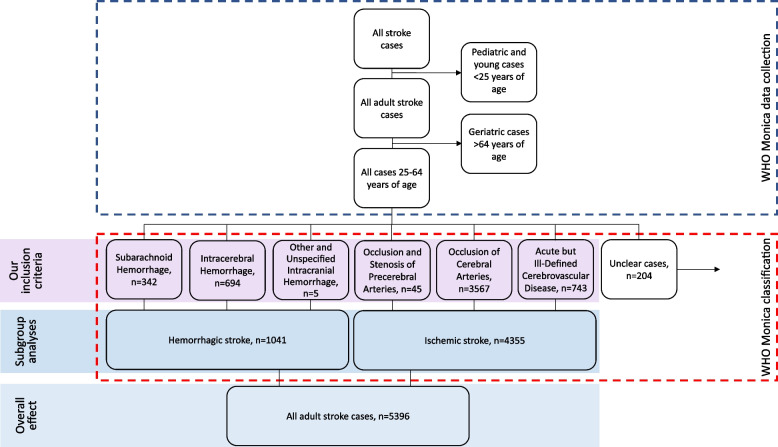
Flow chart of the selection process of eligible cases in both the WHO MONICA study and the current study

The codes for the specific types of stroke were confirmed by specific diagnostic examinations. For subarachnoid hemorrhage (SAH) (ICD-9: 430), autopsy, brain computed tomography (CT), or sampling of cerebrospinal fluid containing blood were required to determine the diagnosis, while for intracerebral hemorrhage (ICD-9: 431), the diagnosis had to be confirmed by CT or autopsy. Ischemic stroke was diagnosed when CT and/or autopsy could verify the infarction and/or exclude hemorrhage and non-vascular disease. In addition to ICD-9 433–434, *acute but ill-defined cerebrovascular disease* (ICD-9: 436) was also classified as ischemic stroke in the WHO MONICA protocol, which was a common practice [20, 21]. These cases mostly died out-of-hospital where definitive diagnostic procedures could not be performed, but the clinical signs observed by the ambulance crew or other previous history indicated that acute stroke was likely. Finally, the WHO MONICA data contained unclassifiable cases (*n* = 204), who had signs of stroke but largely deficient medical records, or residents of Kaunas who had a stroke in medical districts outside Kaunas. We excluded these cases from the current study. Figure 1 shows the flow chart of the selection process of eligible cases in both the WHO MONICA study and the current study. Table S1 shows the ICD-9 codes and the grouping of stroke types in the dataset.

### Exposure assessment

Exposure parameters were calculated for the stroke cases from a continuous time-series of daily mean temperatures in the city of Kaunas which was obtained from the Lithuanian Hydrometeorological Service under the Ministry of Environment. The data are based on measured values of daily temperatures at the Kaunas weather monitoring station, located near the local airport (DMS coordinates 54°53′02.7"N 23°50′09.2"E). This monitoring station corresponds to quality control LST EN ISO 9001:2015 (Certificate No.9000–493). After manually checking the data, we formed three types of frequency distributions of daily temperatures over the study period 1.1.2000–31.12.2015: a) Personal frequency distributions were formed for each individual using the daily temperatures of the days of the hazard period and the 15 reference periods (see below for definitions); b) Season-specific frequency distributions were formed for each of the four seasons using the daily temperatures of all days of the season over the 16 study years; c) Over-annum frequency distributions were formed using the daily mean temperatures of all days of the study period 1.1.2000–31.12.2015. A cold day was then defined as a day with temperature below the 5^th^ percentile of the respective frequency distribution of daily temperatures, resulting in threshold temperatures for a) personal; b) season-specific, and; c) over-annum cold days. The personal cold day definition, with daily mean temperature as the temperature measure, was used *a priori* in the main analyses of this study, while the other definitions were used *a priori* in sensitivity analyses. Essentially, the method of defining personal cold spells using the days of the hazard and reference periods identifies days that are unusually cold for the given time and place [12].

### Statistical analyses

We assigned to each case a one-week hazard period, including the date of the stroke and the 6 preceding days. The 7-day hazard period including the day of the stroke accommodates both a potential triggering effect and a potential lagged effect of cold weather within the week [11–13]. We then assigned to each case 15 reference periods consisting of the same calendar days of the other study years. The daily temperature values for the dates of the hazard period and the 15 Reference Periods were extracted for each case from the shared time series of weather. Each daily value was then compared with the personal threshold temperature of a cold day to define whether the day was statistically cold for the time and place, or not. Pre-specified durations of cold spells were assessed with two mutually complementary methods: a) ≥ 1, ≥ 2, ≥ 3, and ≥ 4 consecutive cold days during the hazard period, with each stratum analyzed separately; b) The absolute number of cold days during the hazard period as a continuous variable, with values from 0 to 7, without the requirement for consecutive order, including all variations in the one model. The decision to use these two complementary models was made *a priori* based on previous evidence to support inference in case of major chance variation [12].

The occurrence of cold spells in the Hazard and Reference Periods formed the contrast for the study. We applied conditional logistic regression using PROC PHREG in SAS, applying the discrete logistic model and forming a stratum for each case. The Odds Ratio (OR) with 95% confidence interval (95% CI) was used as the measure of effect. Long-time trends were controlled by an indicator variable of 4-year intervals over the study period. Season, month, weekday, and holidays were controlled by design. A conscious decision was made not to adjust the models for air pollutants [22].

We conducted stratified analyses according to the season of stroke. We used calendar time to define the 4 seasons (autumn: September to November; winter: December to February; spring: March to May; summer: June to August). We performed subgroup analyses by the ischemic or hemorrhagic etiology of the stroke, age (25–54, 55–64 years) [16, 21], and sex. The z-test was used to test differences between log odds ratios with the following equation:

$$\mathrm{equation}:Z=({E}_{1}-{E}_{2})/\sqrt{{SE({E}_{1})}^{2}+{SE({E}_{2})}^{2}}$$

where Z denotes the Z-test; *E*_1_ and *E*_2_ are the effect estimates (i.e. ln (RR)) of two subgroups; *SE*(E_1_) and *SE*(E_2_) are corresponding Standard Errors of *E*_1_ and *E*_2_ [23].

### Sensitivity analyses

Several sensitivity analyses were performed to assess the robustness of the results. First, instead of using personal frequency distributions of temperature, we used the season-specific and over-annum frequency distributions of daily temperatures in defining cold weather. The latter method corresponds with the traditional method of defining cold spells [24]. Second, we repeated the main analyses using daily minimum and maximum, instead of daily mean temperature in the definition of cold spells. Third, we stratified the data into four 4-year periods, in which the reference periods for each case were limited to the years of the respective 4-year period of their stroke. Fourth, we repeated the analyses of the pre-specified sex and age groups using all cold spell definitions to ensure these results remain robust. Fifth, instead of excluding the unclassifiable cases of stroke (*n* = 204), we included all 5600 consecutive stroke patients from the Kaunas stroke register for the analyses, and also provided estimates for the unclassifiable group.

All analyses were conducted with SAS (SAS, V.9.4; SAS Institute).

## Results

The WHO MONICA register in Kaunas included a total of 5600 adult cases of stroke during the 16-year study period. 204 of these were unclassifiable and were excluded from this study (Fig. 1). A total of 5396 cases satisfied our eligibility criteria. 4355 (80.7%) of these cases experienced an ischemic stroke, while 1041 cases (19.3%) experienced a hemorrhagic stroke. Table 1 shows the characteristics of the study population, with no marked differences in the incidence of stroke by season. Table S2 shows the characteristics over time. Table 2 shows the descriptive statistics of daily outdoor temperature in Kaunas during the study period.

**Table 1:** Characteristics of the eligible cases of stroke in Kaunas, Lithuania, during the study period 2000–2015, by season

**Characteristic**	**Autumn, n (%)**	**Winter, n (%)**	**Spring, n (%)**	**Summer, n (%)**	**All, n (%)**
All cases	1399 (100)	1443 (100)	1373 (100)	1181 (100)	5396 (100)
Ischemic stroke	1111 (79.4)	1166 (80.8)	1129 (82.2)	949 (80.4)	4355 (80.7)
Hemorrhagic stroke	288 (20.6)	277 (19.2)	244 (17.8)	232 (19.6)	1041 (19.3)
Men, all	799 (100)	804 (100)	758 (100)	684 (100)	3045 (100)
Men, ischemic stroke	654 (81.9)	648 (80.6)	635 (83.8)	549 (80.3)	2486 (81.6)
Men, hemorrhagic stroke	145 (18.1)	156 (19.4)	123 (16.2)	135 (19.7)	559 (18.4)
Women, all	600 (100)	639 (100)	615 (100)	497 (100)	2351 (100)
Women, ischemic stroke	457 (76.2)	518 (81.1)	494 (80.3)	400 (80.5)	1869 (79.5)
Women, hemorrhagic stroke	143 (23.8)	121 (18.9)	121 (19.7)	97 (19.5)	482 (20.5)
25–54 years, all	523 (100)	515 (100)	530 (100)	471 (100)	2039 (100)
25–54 years, ischemic stroke	378 (72.3)	391 (75.9)	405 (76.4)	357 (75.8)	1531 (75.1)
25–54 years, hemorrhagic stroke	145 (27.7)	124 (24.1)	125 (23.6)	114 (24.2)	508 (24.9)
55–64 years, all	876 (100)	928 (100)	843 (100)	710 (100)	3357 (100)
55–64 years, ischemic stroke	733 (83.7)	775 (83.5)	724 (85.9)	592 (83.4)	2824 (84.1)
55–64 years, hemorrhagic stroke	143 (16.3)	153 (16.5)	119 (14.1)	118 (16.6)	533 (15.9)

**Table 2:** Descriptive statistics of outdoor temperature (T) in Kaunas, Lithuania, during the study period 2000–2015

**Statistic**	**Autumn, °C**	**Winter, °C**	**Spring, °C**	**Summer, °C**	**All seasons, °C**
Mean (SD)	7.91 (5.24)	-2.49 (5.81)	7.36 (6.47)	17.68 (3.26)	7.66 (8.90)
Range	34.6	35.4	38.5	18.5	52.3
Lowest	-11.6	-25.2	-15.1	8.6	-25.2
Highest	23	10.2	23.4	27.1	27.1
5^th^ percentile threshold	-0.8	-13.9	-4.0	12.6	-7.6
Quartile					
Q1	4.2	-5.7	2.9	15.4	1.2
Q2	7.8	-1	7.6	17.5	7.8
Q3	12.1	1.5	11.9	19.8	15

According to conditional logistic regression, there were positive associations between personal cold spells and stroke. Each additional cold day during the hazard period increased the risk of stroke by 3% (OR 1.03; 95% CI 1.00–1.07). Similar trends of an increased risk with longer episodes of cold weather were observed in the stratified analyses (Table 3, Table S3), although there was some heterogeneity in these estimates. In the season-specific analyses, spring and summer showed positive associations between cold weather and stroke (Table 4). The risk of stroke increased by 8% (OR 1.08; 95% CI 1.00–1.16) per each additional cold summer day.

**Table 3:** Associations between personal cold spells and stroke in Kaunas, Lithuania, 2000–2015, by stroke type. The effect estimates are expressed as Odds Ratios (OR) and their 95% Confidence Intervals (95%CI)

**Exposure**	**All cases, OR (95% CI)**	**Ischemic cases, OR (95% CI)**	**Hemorrhagic cases, OR (95% CI)**
≥ 1 day	1.08 (1.00–1.16)	1.11 (1.02–1.21)	0.96 (0.81–1.14)
≥ 2 days	1.09 (0.99–1.21)	1.13 (1.01–1.27)	0.95 (0.75–1.20)
≥ 3 days	1.16 (0.99–1.37)	1.22 (1.02–1.46)	0.93 (0.63–1.38)
≥ 4 days	1.28 (0.98–1.67)	1.36 (1.01–1.83)	1.00 (0.55–1.83)
Per day	1.03 (1.00–1.07)	1.05 (1.01–1.09)	0.98 (0.91–1.06)

**Table 4:** Associations between personal cold spells and stroke in Kaunas, Lithuania, 2000–2015, by season of stroke

**Season**	**All cases, OR (95% CI)**	**Ischemic cases, OR (95% CI)**	**Hemorrhagic cases, OR (95% CI)**
Autumn	1.00 (0.93–1.07)	1.01 (0.94–1.09)	0.95 (0.82–1.11)
Winter	1.02 (0.97–1.09)	1.04 (0.98–1.11)	0.95 (0.82–1.10)
Spring	1.05 (0.99–1.12)	1.07 (1.00–1.15)	0.97 (0.83–1.14)
Summer	1.08 (1.00–1.16)	1.07 (0.98–1.17)	1.10 (0.93–1.30)

Personal cold spells increased the risk of ischemic stroke (OR 1.05; 95% CI 1.01–1.09) (Table 3). Each additional cold day during the week preceding ischemic stroke increased the risk by approximately 5%, with similar progressive patterns in spring and summer (Table S4). We didn’t find convincing evidence of an association between cold spells and hemorrhagic stroke (Table 3), although a slight signal in the summer season suggests that it may be present at that time of the year (Table 4, Table S4).

Age and sex did not modify the effect of personal cold spells on stroke (Table S5). Results of the sensitivity analyses of the sex and age groups using different cold spell definitions are provided in Tables S6 and S7, where the z-tests also confirmed no effect modification.

The results remained robust when applying the different cold spell definitions and the different temperature measures (Table S8). There was heterogeneity in the stratified analyses by the four 4-year periods for hemorrhagic cases (Table S9), but this may be explained by chance. The inclusion of the unclassifiable cases of the stroke into the analyses did not change the effect estimates in any meaningful way, and the associations for the unclassifiable cases were negative to null (Table S3).

## Discussion

### Main findings

This population-based case-crossover study in Lithuania showed positive associations between personal cold spells and stroke. Each additional cold day in the week before the stroke increased the risk of stroke by 3%. In the summer, each additional cold day increased the risk by 8%. The associations were positive for cold weather and ischemic stroke, but not for hemorrhagic stroke. There was no evidence of effect modification by age or sex. With this exception, all findings were in agreement with our hypotheses. The results remained robust when subjected to a systematic pattern of sensitivity analyses.

### Validity of the results

The study has several limitations. A limitation of the study is that even though the Kaunas population-based stroke register is prospective in nature, it captured cases after a time delay. It is possible that some cases occurring in Kaunas during the years 2000–2015 were not captured by this approach. However, given the extensive data collection protocol, this number is likely to be so small that it does not influence our results. A limitation of the study is classifying *acute but ill-defined cerebrovascular disease* (ICD-9: 436) as ischemic stroke, as this is known to introduce some misclassification in the data [20]. However, this method was part of the WHO MONICA protocol and also a common procedure at the time [21]. Another limitation of the study is that our data did not include cases aged ≥ 65 and thus the assessment of effect modification by age is not comprehensive. A limitation of the study is that we investigated a rare exposure in a relatively small study population, which made statistical inference challenging. We compensated for this by applying two complementary analytical approaches *a priori*, and executed a systematic pattern of sensitivity analyses. A limitation of the study was that it was not possible to ascertain whether the cases had been in the area represented by the Kaunas weather station during the week preceding the stroke. Considering that all eligible cases were *de facto* in the catchment area of the relevant hospital just before the stroke, bias is unlikely. These types of epidemiological studies are blind to the actual individual exposures unless activity diaries and personal monitoring systems are used [24]. The study could be criticized for not analyzing the role of other meteorological factors such as relative humidity or air pressure. However, in previous studies, the role of these factors has been minor compared with temperature [25]. We made a conscious decision not to control the effects of air pollution on stroke. Air pollutants can be treated as intermediate variables in the pathway from cold weather to stroke [22], and adjusting for the intermediate variables would lead to an underestimation of the true overall effect. Air pollution levels in Kaunas are also relatively low, so their role in mediating the effects of cold spells is likely small in this study [26].

The study has several strengths. The prospective study protocol ensured that the case selection procedures and diagnostic criteria remained constant over time. A formal assessment of heterogeneity between effect estimates of different subgroups is another strength. This practice should arguably be routine when estimating and interpreting effect modification in the epidemiological setting. Case-crossover design provides a good platform for studying transient risk factors over time and space, and eliminates many time-invariant and time-varying confounders by design [27]. A strength of the study is the application of personal and seasonal definitions of cold weather. As opposed to the common definition of a cold spell [24], which implicitly captures episodes of low outdoor temperature during the coldest months of the year, this method defines cold weather for the time and place and allows estimation of the relation between the exposure and the outcome for all seasons, not just winter [12, 13]. The strengths of this new approach, including its theoretical and practical implications for environmental epidemiology and public health, have been recently discussed in greater detail [12]. Finally, a strength of the study is the systematic pattern of sensitivity analyses to test the robustness of the results.

### Synthesis with previous knowledge

Several previous studies show associations between low outdoor temperature and stroke [4, 7, 10, 14–18, 21, 28–32]. It remains a rare practice, however, to define outdoor temperature according to the seasonal context in which it occurs [12, 24]. A given outdoor temperature level does not necessarily mean similar personal exposures and similar health effects in different seasons. On the contrary, a given outdoor temperature level can result in different thermal exposures within the personal microclimate when factors such as clothing and indoor heating do not remain constant. An example is being exposed to + 8 °C ambient temperature in winter clothes, versus being exposed to + 8 °C ambient temperature in summer clothes (see also Table 2: the winter maximum and summer minimum temperatures are nearly identical). The latter scenario probably leads to lower indoor temperatures too. Experimental evidence shows that even identical personal exposures can induce different physiological responses in different seasons depending on seasonal and short-term physiological adjustments [33–35]. Our study provides important evidence that the associations between cold weather and stroke are positive in the warm season. To our knowledge, cold summer weather is not recognized as a health risk in any public health programs or guidelines [12].

In our study, we found that cold weather increased the risk of ischemic stroke, but not hemorrhagic stroke. This finding is in contrast with previous evidence. Studies in Australia, South Korea, Hong Kong, and China have reported that cold weather increased the risk of both ischemic and hemorrhagic stroke [14–16, 18]. It is difficult to speculate on the potential reasons for these differences, especially as the biological model for the associations between cold weather and different types of stroke is incomplete [36]. Cold exposure-induced sympathetic drive, increase in systemic vascular resistance, and increase in central aortic blood pressure have been hypothesized to cause hemorrhagic strokes during acute cold exposure [16, 18, 36]. We are not fully satisfied with this model, which doesn’t seem to take fully into account cerebral autoregulation and neurovascular coupling: after all, the increase in systemic blood pressure caused by ambient cold exposure is relatively modest [37]. Could there be some inflammatory process at play? On the other hand, the well-documented cold exposure-induced pro-thrombotic state is a reasonable model for some ischemic strokes during acute cold exposure [30, 31, 38]. At any rate, differentiating between the stronger manifestation of risk factors (e.g. high blood pressure, changes in serum lipid profile, the onset of atrial fibrillation) and the actual causal agents in multi-causal pathways from cold exposure to stroke is not easy. It is interesting that our data signaled some potential associations between cold weather and hemorrhagic stroke in the summer season, but these could not be confirmed. This finding is worthy of further investigation, and could perhaps in the future provide a clue for the related pathomechanics.

Previous evidence is inconsistent on whether or not the effect of cold weather on stroke is modified by sex [7, 17, 18]. Two previous studies conducted in Japan and South Korea reported that the association between cold spells and stroke was stronger among women than men [7, 17], while a recent study from China showed that the association between cold spells and stroke was stronger among men [18]. However, these studies didn’t perform formal heterogeneity testing. Our study did not find convincing evidence of effect modification by sex. The fact that the point estimates for men and women may have looked slightly different in the sensitivity analyses (Tables S6-S7 for ischemic and hemorrhagic stroke) shows the usefulness of formal heterogeneity testing in interpreting effect modification.

We performed two sets of analyses to elaborate potential effect modification by age. First, we produced effect estimates for the pre-specified age groups 25–54 and 55–64 and formally assessed heterogeneity using the z-test: estimates were produced for all eligible cases, for those with ischemic stroke, and for those with hemorrhagic stroke, with no evidence of effect modification (Table S5). Second, we repeated the above-mentioned analyses with alternative definitions of cold spells, with a very subtle signal that the risk could be greater in those aged 25–54, but again with negative z-tests (Tables S6-S7). To summarize, we did not find convincing evidence of effect modification by age. Previous evidence of effect modification by age is inconsistent [4, 7, 14–16, 18], making this one of the important open questions for future studies.

## Conclusions

This population-based case-crossover study in Kaunas provided evidence that there is an association between personal cold spells and stroke. Each additional cold day in the week before the stroke increased the risk of stroke by 3%. The associations were positive for ischemic stroke but not for hemorrhagic stroke. There was no convincing evidence of effect modification by sex or age. Perhaps the most important finding was a positive association between cold weather and stroke in the summer season, meaning that cold summer weather may be a previously unrecognized determinant of stroke. More evidence is needed on the associations between cold weather and different types of stroke in the season-specific context to guide decision-making, policy, and clinical practice.

## Supplementary Information

**Additional file 1: Table S1.** Main diagnoses of stroke according to the International Classification of Diseases (ICD) 9^th^ revision, and the number of cases in each diagnosis group. **Table S2.** Characteristics of the eligible cases of stroke in Kaunas, Lithuania, during the study years 2000–2015, by season and time period. **Table S3.** Sensitivity analyses of the associations between personal cold spells and stroke including the unclassifiable cases (*n*=204) in the Kaunas stroke register, expressed as odds ratios (OR) and 95% confidence intervals (95% CI). **Table S4. **Supporting information of the season-specific trends in overall (analyzing all 5396 cases), ischemic (*n*=4355) and hemorrhagic (*n*=1041) stroke risk associated with prolonged personal cold spells, expressed as odds ratios and 95% confidence intervals. **Table S5.** Associations between personal cold spells and stroke in Kaunas, Lithuania, 2000-2015, by sex and age. **Table S6.** Results of the sensitivity analyses of the associations between season-specific cold spells and stroke by sex and age using the season-specific cold spell definition, expressed as odds ratios and 95% confidence intervals. **Table S7.** Results of the sensitivity analyses of the associations between traditional cold spells and stroke by sex and age using the over-annum cold spell definition, expressed as odds ratios and 95% confidence intervals. **Table S8.** Sensitivity analyses using the different methods of defining cold spells, expressed as odds ratios (OR) and 95% confidence intervals (95% CI). The analyses include all cases meeting the eligibility criteria of the current study (*n*=5396). **Table S9. **Sensitivity analyses of the associations between personal cold spells and stroke by the four 4-year periods, where the personal reference periods are limited to the 4-year period of the stroke, expressed as odds ratios (OR) and 95% confidence intervals (95% CI). The analyses include all cases meeting the eligibility criteria of the current study (*n*=5396).

## Data Availability

The health data was obtained from the Institute of Cardiology under the Lithuanian University of Health Sciences. The data is not accessible online. The weather data was obtained from the Lithuanian Hydrometeorological Service. The data is not accessible online. Data requests can be emailed to: dalia.luksiene@lsmuni.lt. Computing code requests can be emailed to: niilo.ryti@oulu.fi.

## Abbreviations

OR: Odds ratio
CI: Confidence interval
WHO: World health organization
MONICA: Monitoring of trends and determinants in cardiovascular disease
ICD: International Classification of Diseases
SAH: Subarachnoid hemorrhage
CT: Computed tomography
