# Supplementary material for: Risk of ischemic and hemorrhagic stroke in relation to cold spells in four seasons
Source: BMC Public Health. 2023 Mar 23;23:554. doi: 10.1186/s12889-023-15459-4 (PMC10037875; doi:10.1186/s12889-023-15459-4)
Supplement: Supplementary file 1 — Additional file 1: Table S1. Main diagnoses of stroke according to the International Classification of Diseases (ICD) 9th revision, and the number of cases in each diagnosis group. Table S2. Characteristics of the eligible cases of stroke in Kaunas, Lithuania, during the study years 2000–2015, by season and time period. Table S3. Sensitivity analyses of the associations between personal cold spells and stroke including the unclassifiable cases (n=204) in the Kaunas stroke register, expressed as odds ratios (OR) and 95% confidence intervals (95% CI). Table S4. Supporting information of the season-specific trends in overall (analyzing all 5396 cases), ischemic (n=4355) and hemorrhagic (n=1041) stroke risk associated with prolonged personal cold spells, expressed as odds ratios and 95% confidence intervals. Table S5. Associations between personal cold spells and stroke in Kaunas, Lithuania, 2000-2015, by sex and age. Table S6. Results of the sensitivity analyses of the associations between season-specific cold spells and stroke by sex and age using the season-specific cold spell definition, expressed as odds ratios and 95% confidence intervals. Table S7. Results of the sensitivity analyses of the associations between traditional cold spells and stroke by sex and age using the over-annum cold spell definition, expressed as odds ratios and 95% confidence intervals. Table S8. Sensitivity analyses using the different methods of defining cold spells, expressed as odds ratios (OR) and 95% confidence intervals (95% CI). The analyses include all cases meeting the eligibility criteria of the current study (n=5396). Table S9. Sensitivity analyses of the associations between personal cold spells and stroke by the four 4-year periods, where the personal reference periods are limited to the 4-year period of the stroke, expressed as odds ratios (OR) and 95% confidence intervals (95% CI). The analyses include all cases meeting the eligibility criteria of the current study (n=5396). [file 12889_2023_15459_MOESM1_ESM.docx]

**SUPPLEMENTARY MATERIALS**

**Risk of ischemic and hemorrhagic stroke in relation to cold spells in four seasons**

Table S1. Main diagnoses of stroke according to the International Classification of Diseases (ICD) 9^th^ revision, and the number of cases in each diagnosis group

| **ICD-9** | **Type of stroke** | **Cases, N (%)** | **Defined as** | **Total, N (%)** |
| --- | --- | --- | --- | --- |
| 430 | Subarachnoid Hemorrhage | 342 (6.3) | Hemorrhagic stroke | 1041 (19.3) |
| 431 | Intracerebral hemorrhage | 694 (12.9) |  |  |
| 432 | Other and Unspecified Intracranial Hemorrhage | 5 (0.1) |  |  |
| 433 | Occlusion and Stenosis of Precerebral Arteries | 45 (0.8) | Ischemic stroke | 4355 (80.7) |
| 434 | Occlusion of Cerebral Arteries | 3567 (66.1) |  |  |
| 436 | Acute but Ill-Defined Cerebrovascular Disease | 743 (13.8) |  |  |

Table S2. Characteristics of the eligible cases of stroke in Kaunas, Lithuania, during the study years 2000–2015, by season and time period

| **Characteristic** | **Autumn, n (%)** | **Winter, n (%)** | **Spring, n (%)** | **Summer, n (%)** | **All, n (%)** |
| --- | --- | --- | --- | --- | --- |
| 2000-2003 | 366 (100) | 414 (100) | 369 (100) | 337 (100) | 1486 (100) |
| Ischemic stroke | 278 (76.0) | 334 (80.7) | 313 (84.8) | 273 (81.0) | 1198 (80.6) |
| Hemorrhagic stroke | 88 (24.0) | 80 (19.3) | 56 (15.2) | 64 (19.0) | 288 (19.4) |
| 2004-2007 | 391 (100) | 388 (100) | 365 (100) | 280 (100) | 1424(100) |
| Ischemic stroke | 300 (76.7) | 297 (76.5) | 286 (78.4) | 218 (77.9) | 1101 (77.3) |
| Hemorrhagic stroke | 91 (23.3) | 91 (23.5) | 79 (21.6) | 62 (22.1) | 323 (22.7) |
| 2008-2011 | 390 (100) | 381 (100) | 388 (100) | 320 (100) | 1479 (100) |
| Ischemic stroke | 325 (83.3) | 322 (84.5) | 333 (85.8) | 268 (83.8) | 1248 (84.4) |
| Hemorrhagic stroke | 65 (16.7) | 59 (15.5) | 55 (14.2) | 52 (16.2) | 231 (15.6) |
| 2012-2015 | 252 (100) | 260 (100) | 251 (100) | 244 (100) | 1007 (100) |
| Ischemic stroke | 208 (82.5) | 213 (81.9) | 197 (78.5) | 190 (77.9) | 808 (80.2) |
| Hemorrhagic stroke | 44 (17.5) | 47 (18.1) | 54 (21.5) | 54 (22.1) | 199 (19.8) |

Table S3. Sensitivity analyses of the associations between personal cold spells and stroke including the unclassifiable cases (n=204) in the Kaunas stroke register, expressed as odds ratios (OR) and 95% confidence intervals (95% CI)

| **Exposure** | **All cases* (n=5396), OR (95% CI)** | **All cases including unclassifiable cases (n=5600), OR (95% CI)** | **Unclassifiable cases (n=204), OR (95% CI)** |
| --- | --- | --- | --- |
| ≥1 day | 1.08 (1.00-1.16) | 1.07 (1.00-1.15) | 0.90 (0.60-1.33) |
| ≥2 days | 1.09 (0.99-1.21) | 1.08 (0.98-1.19) | 0.81 (0.48-1.35) |
| ≥3 days | 1.16 (0.99-1.37) | 1.14 (0.97-1.34) | 0.66 (0.27-1.61) |
| ≥4 days | 1.28 (0.98-1.67) | 1.25 (0.96-1.63) | 0.50 (0.06-4.15) |
| Per day | 1.03 (1.00-1.07) | 1.03 (1.00-1.06) | 0.91 (0.76-1.09) |

^*^All cases refer to cases meeting the eligibility criteria of the current study

Table S4. Supporting information of the season-specific trends in overall (analyzing all 5396 cases), ischemic (n=4355) and hemorrhagic (n=1041) stroke risk associated with prolonged personal cold spells, expressed as odds ratios and 95% confidence intervals

| **Exposure** | **Subgroup** | **Autumn** | **Winter** | **Spring** | **Summer** | **All seasons** |
| --- | --- | --- | --- | --- | --- | --- |
| ≥ 1 day | All cases* | 0.99 (0.85-1.14) | 1.16 (1.00-1.34) | 1.07 (0.93-1.24) | 1.12 (0.96-1.30) | 1.08 (1.00-1.16) |
| ≥ 2 days | All cases | 0.95 (0.78-1.15) | 0.99 (0.81-1.21) | 1.22 (1.00-1.48) | 1.34 (1.07-1.68) | 1.09 (0.99-1.21) |
| ≥ 3 days | All cases | 0.98 (0.69-1.41) | 0.95 (0.71-1.27) | 1.33 (0.99-1.79) | 1.68 (1.15-2.45) | 1.16 (0.99-1.37) |
| ≥ 4 days | All cases | 1.43 (0.83-2.46) | 0.92 (0.59-1.42) | 1.67 (1.02-2.72) | 1.91 (0.74-4.93) | 1.28 (0.98-1.67) |
| Per day | All cases | 1.00 (0.93-1.07) | 1.02 (0.97-1.09) | 1.05 (0.99-1.12) | 1.08 (1.00-1.16) | 1.03 (1.00-1.07) |
| ≥ 1 day | Ischemic cases | 1.02 (0.87-1.21) | 1.25 (1.06-1.47) | 1.07 (0.91-1.26) | 1.12 (0.94-1.32) | 1.11 (1.02-1.21) |
| ≥ 2 days | Ischemic cases | 1.03 (0.83-1.28) | 0.99 (0.79-1.23) | 1.30 (1.05-1.61) | 1.32 (1.02-1.71) | 1.13 (1.01-1.27) |
| ≥ 3 days | Ischemic cases | 0.96 (0.63-1.45) | 0.95 (0.69-1.31) | 1.50 (1.09-2.07) | 1.82 (1.21-2.74) | 1.22 (1.02-1.46) |
| ≥ 4 days | Ischemic cases | 1.24 (0.63-2.43) | 1.02 (0.63-1.63) | 2.09 (1.23-3.56) | 1.67 (0.56-4.98) | 1.36 (1.01-1.83) |
| Per day | Ischemic cases | 1.01 (0.94-1.09) | 1.04 (0.98-1.11) | 1.07 (1.00-1.15) | 1.07 (0.98-1.17) | 1.05 (1.01-1.09) |
| ≥ 1 day | Hemorrhagic cases | 0.86 (0.62-1.19) | 0.80 (0.55-1.16) | 1.08 (0.78-1.50) | 1.13 (0.80-1.60) | 0.96 (0.81-1.14) |
| ≥ 2 days | Hemorrhagic cases | 0.69 (0.44-1.08) | 1.03 (0.65-1.64) | 0.90 (0.56-1.46) | 1.42 (0.88-2.28) | 0.95 (0.75-1.20) |
| ≥ 3 days | Hemorrhagic cases | 1.07 (0.52-2.21) | 0.97 (0.49-1.92) | 0.70 (0.31-1.59) | 1.07 (0.39-2.98) | 0.93 (0.63-1.38) |
| ≥ 4 days | Hemorrhagic cases | 1.91 (0.74-4.93) | 0.53 (0.16-1.81) | 0.50 (0.11-2.23) | 3.00 (0.42-21.30) | 1.00 (0.55-1.83) |
| Per day | Hemorrhagic cases | 0.95 (0.82-1.11) | 0.95 (0.82-1.10) | 0.97 (0.83-1.14) | 1.10 (0.93-1.30) | 0.98 (0.91-1.06) |

^*^All cases refers to cases meeting the eligibility criteria of the current study (n=5396).

Table S5. Associations between personal cold spells and stroke in Kaunas, Lithuania, 2000-2015, by sex and age

| **Subgroup** | **All cases, OR (95%CI)** | **Ischemic cases, OR (95%CI)** | **Hemorrhagic cases, OR (95%CI)** |
| --- | --- | --- | --- |
| Men | 1.03 (0.99-1.08) | 1.04 (0.99-1.09) | 0.98 (0.88-1.09) |
| Women | 1.04 (0.99-1.09) | 1.05 (1.00-1.11) | 0.98 (0.88-1.10) |
| *z-test (p)** | *-0.29 (0.77)* | *-0.26 (0.79)* | *0 (1)* |
| 25-54 years | 1.04 (0.99-1.10) | 1.06 (1.00-1.13) | 0.99 (0.89-1.10) |
| 55-64 years | 1.03 (0.99-1.08) | 1.04 (0.99-1.09) | 0.98 (0.87-1.09) |
| *z-test (p)* | *0.27 (0.78)* | *0.48 (0.63)* | *0.13 (0.89)* |

*^*^* The z-test and p-value were used to test for statistically significant (p<0.05) differences between log odds ratios of the different subgroups within each stroke type

Table S6. Results of the sensitivity analyses of the associations between season-specific cold spells and stroke by sex and age using the season-specific cold spell definition, expressed as odds ratios and 95% confidence intervals

| **Subgroup** | **All cases (n=5396), OR (95% CI)** | **Ischemic stroke, OR (95%CI)** | **Hemorrhagic stroke, OR (95%CI)** |
| --- | --- | --- | --- |
| Men | 1.01 (0.97-1.06) | 1.01 (0.96-1.05) | 1.04 (0.95-1.14) |
| Women | 1.04 (1.00-1.09) | 1.05 (1.00-1.11) | 1.01 (0.91-1.11) |
| *z-test (p)** | *-0.92 (0.36)* | *-1.11 (0.27)* | *0.43 (0.67)* |
| 25-54 years | 1.04 (0.99-1.09) | 1.04 (0.99-1.10) | 1.04 (0.95-1.13) |
| 55-64 years | 1.02 (0.98-1.06) | 1.02 (0.98-1.06) | 1.01 (0.91-1.11) |
| *z-test (p)* | *0.61 (0.54)* | *0.58 (0.56)* | *0.43 (0.67)* |

^*^The z-test and p-value were used to test for statistically significant (p<0.05) differences between log odds ratios of the different subgroups within each stroke type.

Table S7. Results of the sensitivity analyses of the associations between traditional cold spells and stroke by sex and age using the over-annum cold spell definition, expressed as odds ratios and 95% confidence intervals

| **Subgroup** | **All cases (n=5396), OR (95% CI)** | **Ischemic stroke, OR (95%CI)** | **Hemorrhagic stroke, OR (95%CI)** |
| --- | --- | --- | --- |
| Men | 1.03 (0.99-1.07) | 1.03 (0.99-1.08) | 1.01 (0.92-1.10) |
| Women | 1.04 (1.00-1.08) | 1.06 (1.01-1.11) | 0.97 (0.88-1.07) |
| *z-test (p)** | *-0.35 (0.73)* | *-0.88 (0.38)* | *0.60 (0.55)* |
| 25-54 years | 1.04 (0.99-1.09) | 1.08 (1.02- 1.14) | 0.92 (0.83-1.02) |
| 55-64 years | 1.03 (0.99-1.07) | 1.03 (0.99- 1.07) | 1.04 (0.96-1.13) |
| *z-test (p)* | *0.31 (0.76)* | *0.31 (0.76)* | *-1.82 (0.07)* |

^*^The z-test and p-value were used to test for statistically significant (p<0.05) differences between log odds ratios of the different subgroups within each stroke type.

Table S8. Sensitivity analyses using the different methods of defining cold spells, expressed as odds ratios (OR) and 95% confidence intervals (95% CI). The analyses include all cases meeting the eligibility criteria of the current study (n=5396)

| **Cold spell definition** | **T_min_** | **T_mean_** | **T_max_** |
| --- | --- | --- | --- |
| Personal | 1.04 (1.00-1.07) | 1.03 (1.00-1.06) | 1.02 (0.99-1.06) |
| Season-specific | 1.02 (0.99-1.06) | 1.02 (0.99-1.05) | 1.03 (1.00-1.06) |
| Over-annum | 1.03 (1.00-1.07) | 1.03 (1.00-1.06) | 1.02 (1.00-1.05) |

Table S9. Sensitivity analyses of the associations between personal cold spells and stroke by the four 4-year periods, where the personal reference periods are limited to the 4-year period of the stroke, expressed as odds ratios (OR) and 95% confidence intervals (95% CI). The analyses include all cases meeting the eligibility criteria of the current study (n=5396)

| **Period** | **All cases, OR (95% CI)** | **Ischemic cases, OR (95% CI)** | **Hemorrhagic cases, OR (95% CI)** |
| --- | --- | --- | --- |
| 2000-2003 | 1.03 (0.97-1.10) | 1.05 (0.98-1.12) | 0.96 (0.83-1.11) |
| 2004-2007 | 1.03 (0.96-1.09) | 1.04 (0.96-1.11) | 1.00 (0.88-1.14) |
| 2008-2011 | 1.05 (0.99-1.13) | 1.05 (0.98-1.13) | 1.06 (0.89-1.26) |
| 2012-2015 | 1.03 (0.95-1.11) | 1.06 (0.97-1.15) | 0.91 (0.75-1.10) |
